# Science, statistics and surveys: a herpetological perspective

**DOI:** 10.1111/1365-2664.12463

**Published:** 2015-06-11

**Authors:** Richard A. Griffiths, Jim Foster, John W. Wilkinson, David Sewell

**Affiliations:** ^1^ Durrell Institute of Conservation and Ecology School of Anthropology and Conservation University of Kent Canterbury Kent CT2 7NR UK; ^2^ Amphibian and Reptile Conservation 655A Christchurch Road Boscombe Bournemouth, Dorset BH1 4AP UK

**Keywords:** amphibian, citizen science, great crested newt, modelling, population assessment, reptile, survey and monitoring

## Survey data and conservation needs

Bridging the gap between conservation science and conservation practice is a widely acknowledged issue in applied ecology (Hulme 2011). Nowhere is the gap greater than in the area of data collection, analysis and interpretation. Population assessments for conservation are frequently based on traditional practices that use rules of thumb and quasi‐quantitative methods. This means that important decisions that have far‐reaching conservation, and commercial and financial implications are often based on sketchy population assessments. This is particularly problematic for small‐bodied, cryptic animals that have highly seasonal patterns of behaviour tied to prevailing weather conditions.

Amphibians and reptiles are a case in point and illustrate many issues that have wider implications for biodiversity assessment. Despite a resurgence of interest in the conservation of these animals over the past two decades (Gibbons *et al*. 2000), there remain significant challenges in obtaining population data for amphibians and reptiles that are reliable enough to inform conservation decisions. Even in the UK, which has an impoverished herpetofauna that is relatively well studied, surveys are usually based on methods that have changed little over a quarter of a century. For example, great crested newt *Triturus cristatus* populations can be scored by systems based on simple counts (Table 1). When such counts are carried out as part of a licensed survey to inform recommendations for development impact mitigation, there is a requirement to standardize such counts and record such variables as torch power and water turbidity. However, there remains a multitude of site‐specific and survey‐specific variables that can affect such counts, particularly in wider surveys where standardization may not be required (Schmidt 2003). Consequently, many population assessments may more reliably reflect species detectability than actual population status. Numerous statistical tools are now available that take account of detectability, and can provide estimates of population size, population density or population presence–absence (Table 2). Despite the fact that some of these tools have been around for many years, their use remains largely confined to academia.

**Table 1:** Two current methods for assessing the status of great crested newt populations based on simple counts. Peak count system (Nature Conservancy Council 1989): six surveys are conducted during the breeding season, and the maximum number of newts counted by any method is used to classify the population as ‘low’, ‘good’ or ‘exceptional’. Counts from different ponds on the same site are added together to provide a site total. Population density system (Griffiths, Raper & Brady 1996): newts are counted using one of three methods around the accessible shoreline of a pond, and the density estimated as ‘no. newts per 2 m of shoreline’

Survey method	Low	Good	Exceptional
Peak count method			
Seen or netted by day	<5	5–50	>50
Counted at night	<10	10–100	>100

Survey method	Average	Above average	Good	Excellent
Population density method				
Torch count (clear ponds)	0·67 < 1·88	>1·88 < 2·78	>2·78 < 3·79	>3·79
Torch count (turbid, weedy ponds)	0·32 < 0·74	>0·74 < 1·09	>1·09 < 1·49	>1·49
Trapping	0·51 < 0·96	>0·96 < 1·28	>1·28 < 1·63	>1·63
Netting	0·07 < 0·23	>0·23 < 0·34	>0·34 < 0·46	>0·46

**Table 2:** Summary of methods and modelling tools available for addressing common questions in herpetological population assessments for conservation

Question	Method	Advantages	Disadvantages
How many animals are present at a site?	Capture–mark–recapture modelling	Wide range of software available (e.g. Mark, Capture)	Amphibians and reptiles may be difficult to mark Recapture rates often low Time‐scale may be months or years
What is the relative abundance of the species at a site?	Population densities (counts standardized by survey effort or sampling area)	Can allow rapid population assessments Some software available that accounts for detectability (e.g. Distance)	Amphibian and reptile densities are often low and fluctuate over time Standardization may not account for detectability
	Population indices (counts not standardized by survey effort or sampling area)	Popular, simple method for rapid population assessment Mixture models and *N*‐mixture models can handle replicated counts	Results can be misleading unless they account for detectability Accessible software not yet widely available for practitioners
Where does the species occur?	Presence only/presence–absence at replicated sites across the landscape	Wide range of modelling software available with links to GIS (e.g. Maxent, Presence)	Models using presence‐only data do not account for detectability Simple habitat suitability indices may have unknown reliability
Is it possible to translocate a population?	Depletion/removal modelling	Can determine whether a population is being significantly depleted by removing animals	Appropriate software not yet widely available for practitioners

But are rigorous, statistically defensible population assessments really helpful when it comes to conservation decision‐making? Could their application actually divert resources away from more pressing issues? Exactly what type of evidence is needed for population assessment? In 2011–2012, we held five knowledge exchange workshops in England, Wales and Scotland to explore these issues with professional conservation practitioners. Participants included ecological consultants, planners, local authority ecologists, reserve managers, and government and non‐government agency employees. To ensure discussions remained focussed, habitat assessment and spatial modelling were specifically excluded from the workshops, although nearly all participants considered these to be areas that offered good potential to guide future surveys. Participants were asked to brainstorm what types of survey (i.e. presence–absence, population indices, population densities, population estimates) were needed to guide conservation at different scales. They were then asked to assign priorities for improved design and analysis, and identify possible barriers to implementing changes to current practice. This article explores the main themes that emerged from these workshops, particularly with regard to surveys carried out within professional practice.

## Legal requirements vs. ecological realities

Conservation legislation usually protects individual animals and/or their habitats. However, legislation and guidance are not always framed within ecological language, and consequently, there is no explicit legal obligation to maintain viable populations in the ecological sense. Under the UK Habitats Regulations, for example, there is a legal obligation for the government to achieve and maintain ‘favourable conservation status’ for certain species, but so far it has not been possible to translate this term into meaningful reference values that can serve as benchmarks for ecological monitoring.

When regulations are more explicit in defining legal obligations for protected species in ecological terms, would conservation professionals be able to comply? For example, the UK Habitats Regulations state that species status is favourable when ‘population dynamics data on the species concerned indicate that it is maintaining itself on a long‐term basis as a viable component of its natural habitats’. Given our poor understanding of the ecology and habitat use of these small, cryptic animals, and the time‐scales needed to obtain the necessary data, are we ever likely to know when this is being achieved in a scientific sense? It is hardly surprising, then, that expert opinion is used more often than scientific data. If conservation outcomes can be achieved using simple counts, population indices and surrogate measures, coupled with an expert view of the situation, are detailed population assessments an unnecessary waste of resources?

## Expert knowledge vs. ecological data

Accepting that scientists use language differently to legislators and developers, one role of the ecological consultant is to act as a translator between the two. With traditional statistical methods rooted in fitting models and testing hypotheses using probability theory, there is a significant language barrier that must be overcome. As one consultant pointed out ‘If I tell my client that I can only be 95% certain that a species is absent he will want to know why I can't be 100% certain’. Even if a rigorous population assessment is possible, translating the results of statistical models into meaningful, convincing recommendations that a developer will trust is therefore problematic. However, a concept that is widely understood by developers is that of risk assessment, as it pervades all aspects of project development and execution. If ‘probability’ and ‘best fit’ can be tweaked to reflect ‘risk’ – in particular the risk of breaking the law if a particular protocol is adopted – then part of the language barrier may be overcome. When talking to developers and policymakers, it may therefore be beneficial for scientists to communicate their findings in terms of risk rather than probability.

Ultimately, however, developers – and indeed a lot of conservation decision‐makers – are not interested in statistics or science. What they want is a statement from a competent professional that will allow them to proceed with their activities without breaking either the law or the budget. Whether that statement is based on science or expert opinion is irrelevant to them: if either the science or the opinion is found to be flawed, then the developer can sue the contractor. So what is more trustworthy? All recommendations will rely on an expert view based on evidence, and the importance of incorporating expert opinion into objective decision‐making is increasingly being recognized within conservation. Indeed, tools such as Delphi analysis and protocols for eliciting expert opinions for inclusion within Bayesian models are starting to breach the divide (MacMillan & Marshall 2006; Choy, O'Leary & Mengersen 2009; Kuhnert, Martin & Griffiths 2010). If these methods can be embraced by conservation practice, they may help resolve some of the problems that arise when tight schedules override the importance of obtaining population data.

## Data ownership and transfer issues

The application of occupancy modelling to survey data was largely crystallized by the analytical requirements of the US Geological Survey's Amphibian Research and Monitoring Initiative (MacKenzie *et al*. 2006). Indeed, occupancy modelling lends itself well to the analysis of patchily distributed species such as pond‐dwelling amphibians. However, if such modelling approaches are to be applied on a regional or national basis, centrally coordinated programmes for handling the data are required. Sadly, these are lacking in many countries, including the UK.

Part of the problem is that population assessments are carried out by a variety of practitioners for a variety of purposes. In the UK, for example, the statutory agencies operate licensing systems for ‘European Protected Species’ that theoretically collate data centrally. However, the population data collected under those licences are often incomplete, unstandardized and difficult to access (Edgar, Griffiths & Foster 2005). Species data may – or may not – be deposited in local or regional recording centres, which should then feed into the National Biodiversity Network Gateway system (which is freely accessible). For any aggregated, national analysis, however, conclusions are hamstrung by the fact that data are collected to varying protocols. Surveys are often not effort related, and absences are rarely recorded. There are also issues with patchy coverage: some volunteers are reluctant to deposit data with regional recording centres in case they are sold on for commercial purposes. Equally, some stakeholders retain rights over the data they have collected and are reluctant to release them into the system for dissemination. For strictly protected species in the UK, this issue has been recognized within the licensing system, which now makes submission of records to a local or national scheme a condition. Nevertheless, we still seem some way off from a position whereby any recording scheme can be designed in a way that statistical models can be usefully deployed on a large scale. Unfortunately, problems with data management on a national scale may actually be a global problem (Brown *et al*. 2013; Hill *et al*. 2013; Germano *et al*. 2015). Although NGOs clearly have a significant role to play in improving the situation, leadership in designing and implementing national data management systems must clearly come from government agencies. Without such national long‐term commitment, lessons will not be learned and wheels will continue to be reinvented.

## Building capacity and knowledge exchange

If the gap between science and practice is to be bridged, a reappraisal of existing capacity and knowledge exchange is urgently needed. Universities continue to produce graduates who are short of ecological field skills. On the other hand, professional ecology is failing to take advantage of graduates who emerge with an excellent grounding in statistical ecology and spatial modelling. Many consultants regarded the employment of an ecological statistician or modeller as a low priority. The reasons given for this revolved around the fact that such data analysis is not required within current guidance for population assessments. Although most professionals were positive about the advantages of better survey data analysis, retraining and/or buying in appropriate expertise would require incentives in the form of improved cost‐effectiveness or changes to legislation or guidance. Power analysis has the potential to produce recommendations that can optimize survey effort and improve cost‐effectiveness, but who will pay for it? Government agencies understand the value of such work but are strapped for cash, while funding research for the greater good falls outside the business model for most consultancies.

## Using citizen scientists

Within a generation, herpetological conservation in the UK has evolved from a handful of dedicated naturalists to an army of citizen scientists who do surveys for fun. The National Amphibian and Reptile Recording Scheme (NARRS) relies on willing, trained volunteers surveying predefined grid squares. The data that emerge feed into a national data base that will ultimately produce definitive pictures of status and trends, and circumvent the issues associated with regional biases and data ownership. There is no doubt that the mobilization of this workforce is an outstanding success in the UK and some other countries such as the Netherlands. However, there is a price to pay. Some highly effective survey methods (e.g. trapping for great crested newts) require training and licensing, to ensure that the animal's welfare is not compromised, and are not widely used by volunteers. In the UK, this has resulted in the great crested newt being falsely recorded as absent in many ponds (Sewell, Beebee & Griffiths 2010). Equally, volunteers are less motivated to survey areas where they ‘already know’ the species is absent, or believe the habitat to be unsuitable. Even more problematic is the issue of tagging animals for individual identification for capture–mark–recapture (CMR) modelling. Procedures such as scale clipping and PIT‐tagging may fall under the Animals (Scientific Procedures) Act 1986 and require a licence issued by the UK Home Office. The training, costs and research infrastructure required to obtain a licence may place these methods beyond the reach of volunteers. Clearly, the trade‐offs in using a large number of citizen scientists or a small number of experts deserve close scrutiny when designing surveys.

## What are the priorities for professional practice?

Several widespread species frequently come into conflict with development, resulting in frequent translocations. However, protocols for carrying out such translocations are controversial. Indeed, Reptile Mitigation Guidelines published by Natural England in 2011 were withdrawn just a few weeks later following feedback from some practitioners concerned about recommendations for more thorough surveys. Established guidance exists for great crested newts (English Nature 2001) and states that, as a guide, five nights of trapping with no captures indicate that a reasonable effort has been made to remove all animals from a particular area. Replacing such ‘rules of thumb’ with catch depletion models has strong potential to determine whether the removal is actually effective, and was viewed as a high priority by practitioners at the workshops. In particular, models that include covariates of detection could prove invaluable in reducing the number of exercises that involve ‘false depletions’ due to, for example, periods of inclement weather affecting capture rates.

Capture–mark–recapture models that estimate survival and detectability (e.g. McCrea & Morgan 2014) were viewed as useful tools for obtaining survey‐relevant data. However, their utility as tools for obtaining population size estimates was questionable. Two main issues emerged for this: (i) the relatively intensive survey effort required for obtaining individual capture histories to generate meaningful models; (ii) the usefulness of this type of information for conservation purposes. If a conservation issue arises at a site (e.g. a threat), there may not be enough time to do a comprehensive CMR study. A further issue is that CMR estimates often give wide confidence limits, which raises uncertainties about the precision of estimates. Consequently, some consultants and developers prefer simpler scoring systems for which confidence limits cannot be calculated, despite the fact that they may be even more unreliable! Delays to developments resulting from the discovery of protected species can be extremely costly, and there is often no time to collect the population data required for a reliable assessment of status and viability. Even if there is sufficient time, there are logistical and welfare constraints on individual or batch‐marking of some species for CMR analysis.

The uptake of new methods by professional practice will therefore be strongly influenced by cost, practicality and the explicit requirements of regulatory authorities. Despite their shortcomings, methods based on simple counts are popular, quick and easy and are likely to be here to stay. However, the emergence of *N*‐mixture models that can produce estimates of population size using spatially replicated counts has potential to improve the situation (Royle 2004), and deserves wider attention within professional practice.

## Conclusions

Although science has gone some way to standardize and qualify the different metrics (Sewell *et al*. 2013), we are still some way from having protocols that meet all requirements. Conservation recommendations that stem from population assessments require baseline or control data allowing a measure of population change. Despite several decades of survey effort, establishing such baselines is only just starting to happen in the UK through NARRS.

Among the conservation professionals attending the workshops, views on the application of statistical models to population assessment ranged from the sceptical to the enthusiastic. Although most participants welcomed initiatives to improve data quality and interpretation, this was not universally seen as an overriding priority for progressing survey protocols. Although traditional packages that provide a choice of statistical tools on drop‐down menus carry risks in the hands of the naive user, and are often frowned upon by statistical purists, a user‐friendly interface is exactly what practitioners say they want. It is therefore naive to believe that generalized linear mixed models, information theoretic modelling and Bayesian models will be quickly and widely embraced by ecological practitioners. There again, if the issues concerning data management and flow could be resolved at a national level, it is possible that all the required science could be carried out at a centralized location. However, change is likely to be driven by other factors. With a new generation of graduates raised on R, Matlab and Winbugs entering the job market, the skills required for statistical modelling will eventually pervade ecological practice. Although it can take a long time for science to be translated into legislation, policy and guidance, the drive towards more cost‐effective survey design and analysis will result in closer scrutiny of traditional population assessment protocols. Despite habitat assessment not being included within the workshops, it was widely acknowledged that that this should go hand in hand with population assessments. Indeed, Habitat suitability indices are now widely used to assess the likelihood that an area supports great crested newts (Oldham *et al*. 2000). With computers continuing to fall in price and size while their capacity increases, the potential to capture and disseminate relevant habitat data in a standardized way is enormous. Indeed, ‘presence’ coupled with habitat quality data and expert opinion could be more valuable for conservation than inherently unreliable counts of animals. Recently, there has been substantial industry and government attention on environmental DNA surveys for great crested newts, focused largely on reduced costs and improved reliability over traditional methods (Biggs *et al*. 2015). With the advent of such molecular methods, perhaps the days of counting animals are numbered.

## Data accessibility

Data have not been archived because this article does not contain data.

## Biosketch

**Richard A. Griffiths** and **David Sewell** carry out research on survey methods and the conservation and ecology of amphibians and reptiles. **John W. Wilkinson** runs the National Amphibian and Reptile Recording Scheme and carries out research, monitoring and spatial modelling of distribution patterns. **Jim Foster** oversees species and habitat conservation programmes for the ARC Trust.
